# The relationship between kinaesthesia, motor performance, physical fitness and joint mobility in children living in Nigeria

**DOI:** 10.1186/s12887-023-04348-9

**Published:** 2023-10-23

**Authors:** Ebuka Miracle Anieto, Ijeoma Blessing Anieto, Oluwakemi Adebukola Ituen, Niri Naidoo, Charles I. Ezema, Bouwien Smits-Engelsman

**Affiliations:** 1 Department of Physiotherapy and Paramedicine, School of Health and Life Sciences, Glasgow Caledonian University, Cowcaddens Road, G4 0BA Glasgow, Scotland, U.K.; 2 Department of Health & Rehabilitation Sciences, Faculty of Health Sciences, University of Cape Town, Cape Town, South Africa; 3 Department of Gerontology, Faculty of Social Sciences, University of Southampton, Southampton, United Kingdom; 4 Department of Medical Rehabilitation, University of Nigeria, Enugu Campus, Enugu, Nigeria

**Keywords:** Generalized joint hypermobility, Hypermobility Spectrum Disorder, Kinaesthesia, Motor Performance, Physical fitness, Children

## Abstract

**Purpose:**

This study aimed to determine the relationship between kinaesthesia, motor performance, fitness, and joint mobility in children.

**Methods:**

A descriptive cross-sectional study was conducted involving children from two primary schools in the South-Eastern part of Nigeria. The Beighton criteria were used to measure joint mobility. Motor performance, fitness, and kinaesthesia were measured in all the children. Spearman’s rank correlation was used to evaluate the relationship between the outcomes.

**Results:**

A total of 91 children (51.6% girls) participated in the study. The mean age of the children was 8.20 ± 1.98 years. Using a Beighton score of ≥ 6, Generalized Joint Hypermobility (GJH) was identified in a total of 35 (38.46%) children and was more prevalent in females (60.0%). Joint mobility had significant correlations with most fitness and motor performance items, but not kinaesthesia. Agility & power, and motor performance seem to be reduced if mobility is larger. Kinaesthesia was correlated with most fitness and motor performance items, indicating that better fitness and better motor performance cooccur with better kinaesthesia or vice versa.

**Conclusion:**

Joint mobility may have a significant influence on fitness and motor performance in children. Hence, it may be useful for future studies to investigate how fitness and motor performance modulate the onset and progression of musculoskeletal symptoms in GJH.

**Supplementary Information:**

The online version contains supplementary material available at 10.1186/s12887-023-04348-9.

**What is Known**:

- The prevalence of GJH is higher in the African population compared to Western populations.
- Some children with GJH develop symptoms over time while some others do not.

**What is New**:

- Motor performance and fitness seem to be reduced if joint mobility is larger in children.
- In children, better motor performance and fitness cooccur with better kinaesthesia or vice versa.

## Introduction

Joint hypermobility is characterized by excessive passive and/or active range of motion of the joint beyond normal limits along physiological axes [1]. When multiple joints are involved, it is referred to as Generalized Joint Hypermobility (GJH). In school-aged children, GJH is identified if six or more joints are involved following assessment with the Beighton criteria [2]. GJH is primarily considered as a description of joint mobility and not a disease as it often presents without symptoms [3]. However, some children with GJH may develop musculoskeletal symptoms such as joint pain, joint subluxation, clumsiness, reduced balance, generalized fatigue, reduced kinaesthesia, reduced motor coordination and reduced physical fitness that is not of any rheumatologic, neurologic, or metabolic origin [4, 5, 7–9]. Symptomatic GJH is referred to as Hypermobility Spectrum Disorder (HSD), with a reported prevalence of 17.6% in a population of 10 year-old children [4].The cause of HSD is still unclear, and many clinicians are not conversant with the diagnostic criteria, epidemiology, or clinical features of HSD [5, 6]. However, it is assumed that in the presence of the joint instability, individuals with GJH have a higher risk of developing pain, and joint damage for example dislocations, premature osteoarthritis [7] and abnormal postures given to the abnormal weight-bearing on the joint articular surfaces [8]. This suggests that individuals with joint hypermobility may be needing management as either primary, secondary or tertiary preventive measures [9].

Despite the flexibility that hypermobility offers during movements, some factors may modulate the progression of GJH to HSD, and an early intervention targeted at those factors would be beneficial in preventing the progression of the condition. Several studies have flagged reduced kinaesthesia as a factor that impacts the clinical outcome of individuals with joint hypermobility [10]. Reduced kinaesthesia has also been associated with decreased motor coordination and physical fitness [11]. Studies that have explored the relationship between kinaesthesia, physical fitness, motor performance and joint hypermobility have shown inconsistent results [12–16]. Studies seeking to explore correlations between joint hypermobility and health outcomes in an African context are scarce despite the high prevalence of GJH recorded in Africa [17, 18]. It is important to explore and identify how joint mobility modulate the clinical outcomes of children in an African setting. Exploring these factors will help in establishing indicators to observe in longitudinal studies to identify causality, and in developing interventions that will be specifically targeted at influencing those modulators. Considering that children in Africa have different living and activity patterns compared to Western children, they may not present complaint yet, but that does not rule out the risk of developing complaints as they age. Given the high prevalence of GJH in Nigeria [17, 18], it was important to conduct this study within the Nigerian context. This study therefore explores the relationship between kinaesthesia, motor performance, physical fitness and joint mobility in children, which may provide directions for future longitudinal studies.

## Materials and methods

### Study participants

The respondents for this descriptive cross-sectional study were school-age children within the age bracket of six to eleven years. The children were recruited from one public and one private primary school in Onitsha city, Anambra State, South-Eastern Nigeria. The two schools were randomly selected from a list of primary schools within Onitsha city that was provided by the Anambra State Universal Basic Education Board. The following exclusion criteria were applied:

i) Children who have high risk level and poor safety as it pertains to physical activity. This was assessed using The Physical Activity Readiness Questionnaire (PAR-Q) for children [19]; however, no child was excluded based on the outcome of the questionnaire. The questionnaire was completed by the parents of the children. The criterion for exclusion was if the parents answered ‘yes’ to any of the 7-item questions that are contraindications to physical activity performance [20]. The PAR-Q has been reported as a minimum criterion for determining enrolment into moderate intensity physical activity programmes [21]. PAR-Q is a valid instrument that can be used for all age groups including children [19].
ii) Children who were limited in their ability to understand the testing instructions or the performance of the activities (e.g., cognitive impairment, gross motor impairment etc.).

The study sample size was calculated through a power analysis that showed that a total sample size of 90 is needed for a medium effect size (d = 0.6), at a power of 80%, while alpha is set at 0.05. The G-power analysis software version 3.1 was used for the sample size calculation [22].

### Data collection

The data collection was organized at the premises of the two participating schools during the time allocated for physical education and holiday periods. The headmistress of the schools informed the parents of the children on the dates to bring their children for the tests during the holiday periods. The assessments included anthropometrics and demographics (sex, age, class/grade level, height, weight, body mass index), motor performance and physical fitness using the Performance and Fitness battery (PERF-FIT) [23], kinaesthesia using the wedges [24], and Beighton score [25]. Stations for each test, manned by trained researchers were set up and the children rotated through them in no particular order. The description of the tools used for the tests are provided below.

### Anthropometrics

The measurements included height (cm), weight (kg) and body mass index (BMI). Age-gender specific BMI calculator developed by the National Health Service, United Kingdom was used for the calculation of the children’s BMI centiles [26] and classified as Underweight = ≤ 2nd centile, Normal weight = 3rd to 90th centiles, Overweight = 91st to 97th centiles, Obese = ≥ 98 centile [27].

### Beighton criteria

Joint hypermobility was assessed using the Beighton criteria [25]. The Beighton criterion was chosen because it has the best studied psychometric properties for classifying joint hypermobility and has been validated among children [28]. The Beighton score is comprised of five items, four are passively tested on both sides of the body and one is the active flexion of the trunk. These activities include: (1) the ability to bend the little finger to a right angle (90º) to the back of the hand. (2) the ability to hyperextend the elbows more than 10 degrees. (3) the ability to hyperextend the knees more than 10 degrees. (4) the ability to bend the thumbs back onto the front of the forearm. (5) the ability to put both hands on the floor with both knees held straight. Knee, elbows, and little fingers were measured with a goniometer. The measurement was done by physiotherapists who were trained on goniometric measurements, and it was taken twice for each child (with the average score used) to ensure reliability. One point is given for specific excess joint manoeuvres the child can do. A score of 0–9 was used to divide joint mobility into two categories, normal mobility (0–5) and hypermobility (6–9). We established joint hypermobility with a Beighton score of ≥ 6.

### Motor performance and fitness

The performance and fitness battery (PERF-FIT), which is a valid and reliable assessment battery used to measure motor skill and physical fitness in elementary school aged children within the age bracket of 5–12 years [23] was used. This assessment battery was developed to be both culturally and economically valid for use in low-resourced countries. The assessment battery comprises of two subscales: (a) The performance part: Motor skill subscale made up of 5 skill item series, and (b) Fitness part: Agility and power subscale made up of 5 items. The items in the subscales are described below:

#### Performance part: motor skill subscale

The motor skill subscale or the skill item series (SIS) comprises of five items which include (a) bouncing and catching, (b) throwing and catching, (c) jumping and hopping (d) static balance, (e) dynamic balance. These activities were administered to the children with the difficulty level increased progressively, which is referred to as task-loading. The activities were started with the simplest task and then progressed to the most difficult task within a skill series. The tasks in a particular skill series were discontinued if the child failed to attain the minimum scores for that attempt. A full description of the items and the scoring system for the motor skill subscale is provided as an additional file [see Additional file 1].

#### Fitness part: agility and power subscale

This component is comprised of five items. The items include running, stepping, side-jump, overhead throw and long jump. The activities were demonstrated to the children prior to the test. Each child was given two test trials at a 15 s interval. The best performance of the children during the two trials were then scored and used for the final analysis. A full description of the items and the scoring system for the agility and power subscale is provided as an additional file [see Additional file 1].

### Kinaesthesia

Kinaesthesia was assessed using wedges. Wedges of varying degrees: 1.5º, 3º, 4º, 4.5º, 5º, 6º, 9º, 12º, but equal lengths were positioned on the floor. The use of wedges for assessing kinaesthesia is based on the principle of discrimination of the position of the limbs. A study reported that the use of wedges is a valid outcome instrument for measuring kinaesthesia [24]. The wedges were presented in pairs while the children were in standing position with their eyes covered with a blindfold. This was to prevent the use of visual senses in the discrimination of the limb position. Pairs of wedges with different angles, and two pairs of the same angle were presented for the test. The wedges were presented in a random order. The children were asked to identify the more elevated heel by raising their ipsilateral hand. The children were given one point for correct answers and zero points for incorrect answers. As an additional outcome, a penalty score was given for incorrect answers based on the angle difference. An incorrect answer for the largest wedge difference amounted to the highest penalty score. The scores were then summed up and used for the final analysis.

### Statistical analysis

The descriptive statistics of median, interquartile range, frequency and percentage were used to describe the anthropometric characteristics of the children, their motor performance, physical fitness, kinaesthesia and Beighton score. The normality of the data was evaluated using the Shapiro-Wilks test, which showed that the data were not normally distributed. Therefore, the non-parametric statistical tests were used for the analysis. Fisher’s Exact test was used to determine the gender-specific difference in the prevalence of GJH. The Mann-Whitney U test was used to determine the between-group (hyper and normal mobility) differences in the anthropometrics of the children. Spearman’s rank correlation was used to determine the relationship between the variables of interest (motor performance, physical fitness, kinaesthesia and joint range of motion). Alpha was set at 0.05 (for a 95% confidence level).

## Results

### Characteristics of the participants and prevalence of GJH

**Fig. 1:**
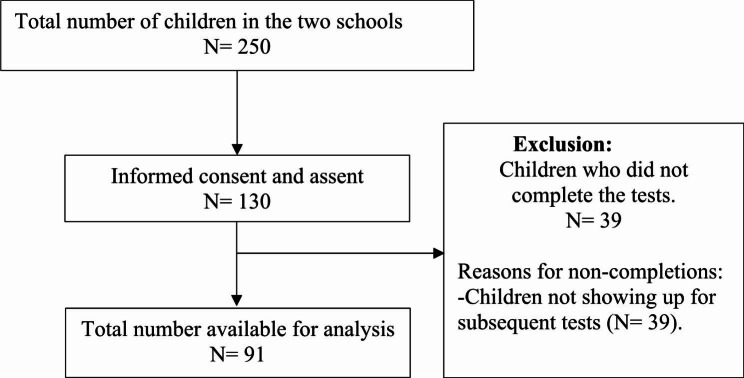
Flow chart of the recruitment process

A total of 91 children completed the tests and their data was used for analysis (Fig. 1). Table 1 shows the demographic and anthropometric characteristics of the children. GJH was identified in a total of 35 (38.46%) children following the Beighton cut-off criteria of the presence of hypermobility in ≥ 6 joints. Even though hypermobility was found in 60% of the females and 40% of the males, the difference did not reach a statistical significance (X^2^ = 1.589, p = 0.281). None of the children in the study presented with pain.

**Table 1:** Characteristics of the participants

Variables Total (n = 91)	Median	IQR
Age (years)	8.20	4.00
Weight (kg)	28.00	17.00
Height (m)	1.31	0.21
BMI (kg/m^2^)	16.12	5.96
Waist circumference	60.00	9.00
**BMI Classification**		
Underweight, n (%)	22	24.2
Normal weight, n (%)	53	58.2
Overweight, n (%)	10	11.0
Obese, n (%)	6	6.6
Gender		
N (Male/Female)	44/47	
% (Male/Female)	48.4/51.6	

IQR: Interquartile range

### Relationship between joint mobility (Beighton score), kinaesthesia, physical fitness and motor performance in children

Joint mobility (Beighton score) had no significant correlation with kinaesthesia (correct wedges, and penalty scores). However, joint mobility (Beighton score) had significant negative correlations with most fitness items; side jump (r = -0.244, p = 0.020), long jump (r = -0.267, p = 0.011), and overhand throw (r = -0.479, p < 0.001), a positive correlation (r = 0.319, p = 0.002) with ladder run (time it takes to complete running in agility ladder), and no significant correlation (r = 0.176, p = 0.114) with ladder steps (time it takes to complete stepping in agility ladder); indicating that more mobility is associated with less side jump, less long jump and overhand throw distance, and longer running time. Similarly, significant negative correlations were found between joint mobility (Beighton score) and most motor performance items; ball bounce (r = -0.318, p = 0.003), ball throw (r = -0.386, p < 0.001), and dynamic balance (r = -0.216, p = 0.044), except static balance (r = -0.155, p = 0.145), and total jump & hop (r = -0.085, p = 0.427); indicating that more mobility is associated with less ball bounce, and ball throw counts, and less dynamic balance.

There were significant positive correlations between kinaesthesia (correct wedges) and some fitness items; side jump (r = 0.293, p = 0.005), and long jump (r = 0.284, p = 0.006), negative correlation with ladder run (r = -0.282, p = 0.007), and no significant correlations with ladder steps (r = -0.184, p = 0.098), and overhand throw (r = 0.188, p = 0.075); indicating that better kinaesthesia cooccur with better side jump, long jump, and ladder run. Significant positive correlations were also found between kinaesthesia (correct wedges) and most motor performance items; ball bounce (r = 0.306, p = 0.004), ball throw (r = 0.253, p = 0.019), and dynamic balance (r = 0.267, p = 0.012), except static balance (r = 0.197, p = 0.063), and total jump and hop (r = 0.169, p = 0.113); indicating that better kinaesthesia cooccur with better ball bounce, ball throw, and dynamic balance. The details of the results are presented in Table 2.

**Table 2:** Correlations between joint mobility (Beighton score), kinaesthesia, running and agility, and motor performance items

Correlations															
			Beighton score N = 91	Correct Wedges N = 91	Penalty scores N = 91	Ladder run N = 91	Ladder steps N = 81	Side jump N = 91	Long jump N = 91	Overhand throw N = 91	Ball bounce N = 91	Ball throw N = 91	Total jump &hop N = 90	Static balance N = 91	Dynamic balance N = 90
	Beighton score	Corr.	1.000	− 0.0138	0.147	0.319	0.176	− 0.244	− 0.267	− 0.479	− 0.318	− 0.386	− 0.085	− 0.155	− 0.216
		Sig.	.	0.193	0.163	*0.002	0.114	*0.020	*0.011	**< 0.001**	*0.003	**< 0.001**	0.427	0.145	*0.044
	Correct Wedges	Corr.	− 0.0138	1.000	− 0.980	− 0.282	− 0.184	0.293	0.284	0.188	0.306	0.253	0.169	0.197	0.267
		Sig.	0.193	.	< 0.001	*0.007	0.098	*0.005	*0.006	0.075	*0.004	*0.019	0.113	0.063	*0.012
	Penalty scores	Corr.	0.147	− 0.980	1.000	0.256	0.215	− 0.295	− 0.284	− 0.198	− 0.324	− 0.264	− 0.149	− 0.177	− 0.286
		Sig.	0.163	< 0.001	.	*0.014	0.052	*0.005	*0.006	0.060	*0.002	*0.014	0.162	0.096	*.007

*Significance (2-tailed), alpha set at 0.05 when analysed with Spearman’s rank correlation

## Discussion

Studies investigating the relationship between kinaesthesia, motor performance, fitness, and joint mobility in children within the African context are scarce. Hence, the study was conducted to add to the body of knowledge and to address the gap in literature.

There was no significant correlation between joint mobility and kinaesthesia in children in our study. This interesting finding challenges the assumption in literature that GJH is synonymous with joint instability and subsequent destruction of mechanoreceptors and impairment of kinaesthesia [2]. It is noteworthy that the instrument used to test kinaesthesia and the position the test is carried out are considered as possible reasons for the different outcome of kinaesthesia in children with GJH. Akkaya et al. [29] tested ankle kinaesthesia in children with GJH in supine position (unloaded position) using a digital goniometer and observed significantly lower kinaesthesia in children with GJH. Whereas Ituen et al. [30] tested ankle kinaesthesia in the loaded position using wedges and found better kinaesthetic sense in children with GJH. Our study tested kinaesthesia in the loaded position using wedges similar to the study by Ituen et al. [30], but with smaller differences between the heights of the wedges. The loaded position is the functional position of the legs and load receptors play important role in the sensory information needed for kinaesthesia/proprioception [31]. This makes the wedges an appropriate instrument to assess kinaesthesia in future cohort studies [30]. Similar to the outcome of our study, Pacey et al. [32] reported no significant correlation between kinaesthesia/proprioception and joint mobility in a study of children with symptomatic joint hypermobility. Their study tested knee kinaesthesia in a loaded position and they concluded that kinaesthesia even in a population of children with symptomatic joint hypermobility is not altered.

In our study, most fitness items on the PERF-FIT were associated with joint mobility, suggesting that fitness may be reduced in children with GJH. The study by Engelbert et al. [16] similarly found a reduction in fitness in children with symptomatic GJH. They inferred that pain and consequent low engagement in exercise leading to deconditioning may explain the decreased physical fitness marked in children with symptomatic GJH [16]. Considering that none of the children in our study presented with pain, it is unlikely that the reduced physical fitness recorded was due to the impact of pain on exercise performance. A study reported that physical fitness was reduced even in individuals with GJH that engaged in routine exercise, which supported the independent association of GJH with reduced physical fitness [33]. The most probable explanation to the reduced physical fitness is the negative impact of connective tissue laxity on active joint stabilization mechanisms [34]. For example, during activities like running that requires high coordination, more demanding adaptive strategies (e.g., co-contraction, extended activation of specific muscle groups) are needed to keep the joints stable in individuals with GJH [35]. Hence, a greater energy demand is needed during activity performance, which may lead to easy fatigability in individuals with GJH [36]. More studies are needed to establish the factors that predispose children with joint hypermobility to fitness deficits.

Most motor performance items on the PERF-FIT negatively correlated with joint mobility, suggesting that motor performance may be reduced in children with GJH. This finding is in agreement with the results from other studies reporting that motor performance is reduced in children with GJH [1, 37, 38]. Possible explanations to the motor deficits recorded in children with GJH include the association of GJH with congenital benign hypotonia [39], reduced muscular strength [40], and reduced physical fitness [33]. Studies have reported that both fine and gross motor skills are usually limited in children with GJH [15, 41], which will make activities like ball throwing & catching, ball bouncing & catching, and dynamic balance to be challenging for the children.

In our study, kinaesthesia had significant positive correlations with most motor performance items suggesting that good motor performance coincides with good kinaesthesia and vice versa. The result is consistent with existing evidence that kinaesthesia is essential for the neural control of human movement, and that kinaesthetic impairment may result to reduced motor control outcomes [42–44]. Similarly, there was a significant positive or negative correlation between kinaesthesia and most agility (fitness) items. The results suggest that kinaesthesia may be a moderating factor for motor performance and fitness in children. The kinaesthetic system is essential in maintaining joint stability [45]. Therefore, kinaesthetic deficits may lead to poor judgement of the body position during movement, and abnormal postures during functional activities [46], which may have impact on motor performance and fitness. However, it should be noted that the observed strength of correlations indicates that kinaesthesia does not explain a lot of the motor performance and fitness in our study. Different living circumstances, pattern of activity, risk of underweight (less muscle mass) observed in the children (which differs from the usual overweight observed in children in Western populations) may also have influences on motor performance and fitness. More rigorous studies (e.g., longitudinal studies and randomized controlled trials) are needed to establish the influence of kinaesthesia on the development of symptoms in individuals with GJH.

### Limitations of the study

The study used a cross-sectional design, which cannot be used to establish cause and effect. Therefore, the results of this study are suggestive and not conclusive. Sufficient time was provided for rest between the various tests, and we also watched out for signs of fatigue, however, given that some of the tests are physically demanding and the number of tests carried out each day, there was a chance of underperformance because of fatigue.

## Conclusions

The study showed that joint mobility (Beighton score) was significantly correlated with most fitness and motor performance items, but not kinaesthesia. A large population-based longitudinal study will be necessary to evaluate how kinaesthesia, motor performance and physical fitness change over time in children with GJH and in children with normal mobility. Despite that our study did not find a correlation between joint mobility and kinaesthesia, there are indications from our study that kinaesthesia may moderate motor performance and physical fitness in children.

### Electronic supplementary material

Below is the link to the electronic supplementary material.

Supplementary Material 1

## Data Availability

The datasets generated and/or analysed during the current study are not publicly available due to confidentiality and privacy considerations but are available from the corresponding author on reasonable request.

## Abbreviations

ANCOVA: Analysis of Covariance
BMI: Body Mass Index
CNS: Central Nervous System
GJH: Generalized Joint Hypermobility
HSD: Hypermobility Spectrum Disorder
NM: Normal Mobility
PAR-Q: Physical Activity Readiness Questionnaire
PERF: FIT Performance and Fitness battery
SIS: Skill Item Series
WHO: World Health Organization
